# Positive Peripheral Social Network Members in Older Age: Global and Daily Appraisals of Social Partners

**DOI:** 10.1093/geroni/igab046.3316

**Published:** 2021-12-17

**Authors:** Claire Growney, Tammy English

**Affiliations:** 1 Washington University in St. Louis, Saint Louis, Missouri, United States; 2 Washington University in St. Louis, St. Louis, Missouri, United States

## Abstract

Socioemotional selectivity theory suggests older adults maintain relationships with close social partners with whom they experience positive emotions. It is unclear how age and closeness predict social partner appraisals in different contexts. We examine semantic and experiential appraisals of positivity, as well as emotional outcomes. Participants (N = 258) aged 25-85 (M = 52.05, SD = 16.31) reported their general experience of enjoyment and conflict with social partners of varying closeness. In an experience sampling procedure (6x/day for 10 days), participants reported their current experience of emotions and information about their most recent social interaction: pleasure, discomfort, and relationship closeness with their social partner. Semantic (global) appraisals of relationships positively predicted experiential (daily) appraisals, and this association was stronger among relatively older adults. Results revealed older adults gave less negative appraisals compared to younger adults, regardless of closeness. Older adults reported more positive appraisals than younger adults for non-close relationships, whereas close relationships were evaluated positively regardless of age. For younger adults, interaction pleasure with non-close partners was less strongly linked to subsequent positive emotions than pleasure with close partners. For older adults, however, interaction pleasure predicted greater subsequent positive emotions regardless of relationship closeness. Overall, these findings suggest older adults’ positive appraisals of partners are not simply the result of emotionally gratifying memory distortions. Older adults may be able to derive emotional benefits from a wider variety of social interactions than younger adults, suggesting peripheral social network members can be leveraged to enhance emotional well-being in later adulthood.

